# Lipoprotein(a) plasma levels are not associated with incident microvascular complications in type 2 diabetes mellitus

**DOI:** 10.1007/s00125-020-05120-9

**Published:** 2020-03-09

**Authors:** Sunny S. Singh, Mardin Rashid, Aloysius G. Lieverse, Florian Kronenberg, Claudia Lamina, Monique T. Mulder, Yolanda B. de Rijke, Eric J. G. Sijbrands, Mandy van Hoek

**Affiliations:** 1 Department of Internal Medicine, Erasmus MC – University Medical Center Rotterdam, Doctor Molewaterplein 40, 3015 GD Rotterdam, the Netherlands; 2 Department of Internal Medicine, Maxima Medical Center, Eindhoven, the Netherlands; 3 Institute of Genetic Epidemiology, Department of Genetics and Pharmacology, Medical University of Innsbruck, Innsbruck, Austria; 4 Department of Clinical Chemistry, Erasmus MC – University Medical Center, Rotterdam, the Netherlands

**Keywords:** Lp(a), *LPA*, Microvascular complications, Nephropathy, Neuropathy, Retinopathy, rs10455872, rs3798220, SNP, Spline

## Abstract

**Aims/hypothesis:**

Microvascular disease in type 2 diabetes is a significant cause of end-stage renal disease, blindness and peripheral neuropathy. The strict control of known risk factors, e.g. lifestyle, hyperglycaemia, hypertension and dyslipidaemia, reduces the incidence of microvascular complications, but a residual risk remains. Lipoprotein (a) [Lp(a)] is a strong risk factor for macrovascular disease in the general population. We hypothesised that plasma Lp(a) levels and the *LPA* gene SNPs rs10455872 and rs3798220 are associated with the incident development of microvascular complications in type 2 diabetes.

**Methods:**

Analyses were performed of data from the DiaGene study, a prospective study for complications of type 2 diabetes, collected in the city of Eindhoven, the Netherlands (*n* = 1886 individuals with type 2 diabetes, mean follow-up time = 6.97 years). To assess the relationship between plasma Lp(a) levels and the *LPA* SNPs with each newly developed microvascular complication (retinopathy *n* = 223, nephropathy *n* = 246, neuropathy *n* = 236), Cox proportional hazards models were applied and adjusted for risk factors for microvascular complications (age, sex, mean arterial pressure, non-HDL-cholesterol, HDL-cholesterol, BMI, duration of type 2 diabetes, HbA_1c_ and smoking).

**Results:**

No significant associations of Lp(a) plasma levels and the *LPA* SNPs rs10455872 and rs3798220 with prevalent or incident microvascular complications in type 2 diabetes were found. In line with previous observations the *LPA* SNPs rs10455872 and rs3798220 did influence the plasma Lp(a) levels.

**Conclusions/interpretation:**

Our data show no association between Lp(a) plasma levels and the *LPA* SNPs with known effect on Lp(a) plasma levels with the development of microvascular complications in type 2 diabetes. This indicates that Lp(a) does not play a major role in the development of microvascular complications. However, larger studies are needed to exclude minimal effects of Lp(a) on the development of microvascular complications.

**Electronic supplementary material:**

The online version of this article (10.1007/s00125-020-05120-9) contains peer-reviewed but unedited supplementary material, which is available to authorised users.

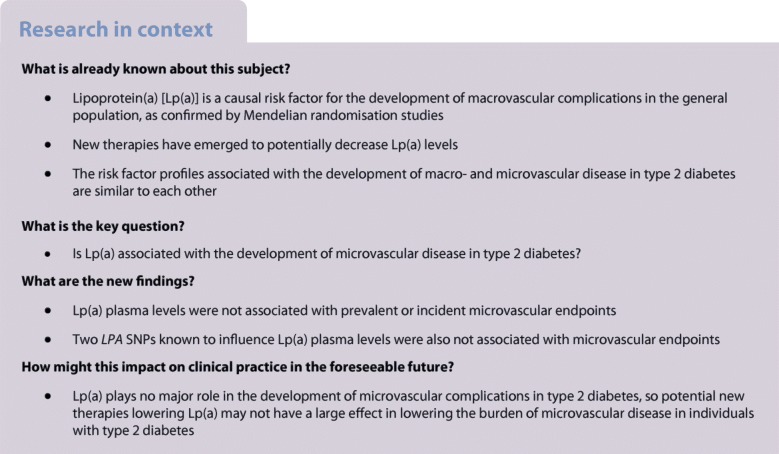



## Introduction

Microvascular complications greatly reduce the quality of life of individuals with type 2 diabetes. Microvascular damage causes end-stage renal disease, blindness and peripheral neuropathy [1–3]. Strict control of risk factors (e.g. lifestyle, hyperglycaemia, hypertension and dyslipidaemia) reduces the incidence of microvascular complications. However, even when people with type 2 diabetes receive optimal treatment according to the current standards, there is a residual risk of development and progression of complications [4, 5]. Thus, there is a need to identify additional risk factors. A better understanding of these factors paves the way for better prediction, prevention and treatment. Lipoprotein(a) [Lp(a)] is a known strong risk factor for macrovascular disease in the general population [6]. New therapies have emerged that reduce Lp(a), and thereby could potentially decrease the burden of macrovascular disease [7]. Microvascular disease in type 2 diabetes could be a potential new target for Lp(a)-lowering therapies. Lp(a) has been related to atherosclerosis and macrovascular disease [6, 8]. Microvascular diseases share underlying mechanisms with atherosclerosis and macrovascular disease [9, 10]. We hypothesised that Lp(a) is a risk factor for microvascular disease in type 2 diabetes.

Lp(a) is an LDL-like lipoprotein with an apolipoprotein B-100 molecule, to which a unique apolipoprotein A (ApoA) is attached via a disulfide bond. Lp(a) is considered to be a more pro-thrombotic and atherogenic particle than LDL. Lp(a) plasma levels are highly heritable and are associated with the size of the ApoA protein, which is determined by the number of kringle IV type 2 (KIV) repeats [11]. Furthermore, two well investigated *LPA* SNPs, rs10455872 and rs3798220 influence Lp(a) plasma levels, and are associated with a lower number of Lp(a) KIV repeats [12]. In prospective, population-based studies, positive associations between Lp(a) plasma levels and macrovascular disease have been reported [13, 14]. These findings have been confirmed using the Mendelian randomisation approach, which indicates a causal role of Lp(a). Lp(a) levels ≥30 mg/dl are associated with macrovascular disease. The Copenhagen City Heart Study showed a 1.6-fold increased risk for incident myocardial infarction for Lp(a) levels between 30 and 76 mg/dl, corresponding to the 67th to 90th percentile, this further risk increased with higher Lp(a) levels [15]. Findings on the relationship of Lp(a) levels and microvascular complications in type 2 diabetes have been conflicting [16–27]. Most of these studies were of Asian populations and the generalisability of the findings to other ethnicities remains uncertain. Regarding diabetic retinopathy, two cross-sectional studies and one prospective study found that high Lp(a) levels significantly associate with diabetic retinopathy [16, 17, 23]. However, other cross-sectional studies did not find any association [20, 22]. In diabetic nephropathy, two small prospective studies found Lp(a) to be positively associated with nephropathy [19, 24], whereas another prospective study by Lin et al [25] did not find any association. Similarly, the few reports for diabetic neuropathy are also conflicting [26, 27]. The association of known *LPA* SNPs and ApoA isoforms with microvascular complications in type 2 diabetes has never been investigated. We therefore investigated the association of Lp(a) plasma levels and selected *LPA* SNPs with prevalent and incident microvascular complications of type 2 diabetes using data from the DiaGene study, a prospective case−control study on type 2 diabetes with follow-up on microvascular complications [28].

## Methods

### Study design

The overall aim of the DiaGene study is to unravel the aetiology of type 2 diabetes and its complications, by identifying risk factors, e.g. genomic, glycomic and lipidomic factors. The design of the DiaGene study has been reported elsewhere [28]. Briefly, the DiaGene study is an all lines of healthcare prospective case−control study collected of individuals resident in and around the city of Eindhoven in the Netherlands. All hospitals in this area participated, as well as the centre for primary care diagnostics. Hence, virtually all individuals with type 2 diabetes in this area were approached for inclusion. Initially, 2065 patients were included by physicians at all centres. Of these, 179 patients were excluded. Reasons for exclusion were: no diabetes, type 1 diabetes, maturity-onset diabetes of the young, latent auto-immune diabetes in adults, double inclusion of the participant, post-pancreatitis diabetes, withdrawal of consent during the study period and missing informed consent. Finally, 1886 patients with type 2 diabetes and 854 controls were included in the DiaGene study, which consisted mostly of people of European descent. Written informed consent was obtained from all participants. This study was approved by the medical ethics committees of the Erasmus MC and the local hospitals in Eindhoven. For the analyses described in this paper, only data on type 2 diabetes cases were analysed.

### Clinical data

Laboratory data and anthropometrics were derived from medical records at inclusion. By means of a questionnaire, medical history, family history and lifestyle information were collected. Mean arterial pressure (MAP) was calculated with the formula (2 × diastolic pressure + systolic pressure)/3. Non-HDL-cholesterol was calculated with the formula non-HDL-cholesterol = total cholesterol – HDL-cholesterol.

### Definitions of microvascular complications

Diabetic retinopathy was scored by ophthalmologists as absent or present, according to the Dutch guideline for diabetic retinopathy [29]. If present, it was classified as non-proliferative, proliferative, treated with photo-coagulation or treated with intra-vitreal injections. Neuropathy was diagnosed by a podiatrist, neurologist or the treating physician and graded according to the Sims classification [30]. Neuropathy data were only available for those individuals with type 2 diabetes who were under surveillance in outpatient clinics (*n* = 796). Nephropathy was defined as microalbuminuria (ACR ≥2.5 mg/mmol for men or ≥3.5 mg/mmol for women) at two of three consecutive measurements, or when high microalbuminuria or macroalbuminuria was present at one measurement (ACR ≥12.5 mg/mmol for men and ≥17.5 mg/mmol for women) [28].

### Lp(a) plasma concentration and Lp(a) SNPs

Venous blood samples were drawn from individuals with type 2 diabetes at enrolment; after centrifugation, the plasma and buffy coat were separated. Plasma samples were directly stored at –80°C. Lp(a) concentrations were measured in samples that had not been defrosted previously. Lp(a) concentrations in plasma were measured with the KIV-2 number-independent Randox immunoassay on a Roche Cobas c501 Chemistry Analyzer [31, 32], with an Lp(a) concentration range of 3–300 mg/dl. A total of 1850 type 2 diabetic individuals with successfully measured Lp(a) plasma levels were included in the analyses. *LPA* SNPs rs10455872 and rs3798220 were genotyped by using Taqman allelic discrimination assays designed and optimised by Applied Biosystems (Foster City, CA, USA). The *LPA* SNPs rs10455872 and rs3798220 were successfully genotyped for 1696 (90%) and 1727 (92%) participants, respectively. Reactions were performed on the Taqman Prism 7900 HT platform.

### Statistical analysis

To compare baseline variables, the independent samples *t* test was used for continuous variables with a normal distribution and the Mann–Whitney test was used for continuous variables with a non-normal distribution. Normality was assumed when skewness and kurtosis were within the range of −1 and +1. For categorical variables, the χ^2^ test was applied. To assess the relationship between Lp(a) and microvascular endpoints, we used Lp(a) plasma levels as an independent primarily binary variable (<30 mg/dl and ≥30 mg/dl). This cut-off level is based on clinical studies in the general population, where an almost two times higher risk of a major adverse cardiovascular event is present when Lp(a) plasma levels exceed 30 mg/dl [15]. However, specifically for stroke, the ‘Emerging risk factors consortium’ observed a higher threshold of Lp(a) concentrations [14]. In addition, we assessed the relationships of the microvascular complications with Lp(a) as a continuous variable and in categorical quartiles. For prevalent microvascular complications at baseline, logistic regression was used to assess the relationship of Lp(a) and each of the microvascular complication endpoints. Cox proportional hazards models were used to prospectively assess the association between Lp(a) as an independent variable and each microvascular complication endpoint as a dependent variable. The prevalent cases at baseline were excluded from the prospective analyses. In addition, to assess the association of Lp(a) with kidney function, estimated glomerular filtration rate (eGFR) determined by the Modification of Diet in Renal Disease (MDRD) study equation [33], we performed linear regression, with % difference in eGFR as the dependent variable and Lp(a) the independent variable. The % difference in eGFR was calculated using the formula ([eGFR_baseline_ – eGFR_follow-up_]/eGFR_baseline_) × 100. Model 1 was adjusted for sex and age. Model 2 was adjusted for the following additional risk factors for microvascular complications: MAP, non-HDL-cholesterol, HDL-cholesterol, HbA_1c_, BMI, duration of type 2 diabetes and smoking. In order to detect whether there was any other form of relationship we may have missed by analysing Lp(a) linearly or in quartiles, we performed non-linear spline analyses. More specifically, we applied P-splines (penalised cubic B-splines) in both the logistic regression as well as Cox models [34]. This involves selecting a high number of equidistant knots, followed by a penalty term, which is optimised via generalised cross validation to avoid overfitting. This is a data-driven and explorative approach for detecting any non-linear relationships. Models 1 and 2 described above were also applied to assess the relationship between the SNPs and each microvascular endpoint. For analysing this relationship between the SNPs rs10455872, rs3798220 and microvascular endpoints an additive genetic model was used. To assess if *LPA* SNPs carrier status associates with Lp(a) plasma concentration, one-way ANOVA was applied. We tested the Hardy–Weinberg equilibrium before incorporating the *LPA* SNPs in the analyses. A *p* value of <0.05 was considered statistically significant. Statistical analyses were performed using SPSS 25.0 (IBM, Armonk, NY, USA). Non-linear spline analyses were performed using R version 3.6.0 (https://www.r-project.org/), including packages ‘mgcv’ and ‘survival’. For cross-sectional and prospective analyses concerning Lp(a) as binary predictor, we had 80% power to detect an OR of 1.4 and an HR of 1.5 for each microvascular endpoint, respectively. And for cross-sectional and prospective analyses with Lp(a) as continuous predictor, we had 80% power to detect an OR of 1.09 and an HR of 1.013 for each microvascular endpoint, respectively. Moreover, for genetic analyses, we had 80% power to detect an HR of 1.7 for each separate SNP (rs10455872 and rs3798220) separately. ESM Table 1 provides an overview of conducted post hoc power analyses [35–37].

## Results

### Baseline characteristics of the study population

The baseline characteristics and the number (and percentage) of individuals with data missing per characteristic of the study population are shown in Table 1. The age of the participants ranged from 27 to 94 years, with a mean age of 65.2 years; 46% were female. The mean duration of type 2 diabetes before the baseline investigation was 10.02 years, mean duration of follow-up from baseline was 6.97 years. In the total population, mean Lp(a) was 27.40 mg/dl and the median was 11.00 mg/dl. ESM Table 2 shows the Lp(a) median concentration according to each of the microvascular complications. The prevalence of retinopathy, nephropathy and neuropathy was 17%, 23% and 31%, respectively (Table 1). The incidence of retinopathy, nephropathy and neuropathy was 16%, 19% and 49.2%, respectively. Of note, data regarding the prevalence and incidence of neuropathy were available only for the 796 individuals who attended the outpatient clinic. Among the individuals free of a microvascular complication at baseline, those who developed microvascular complications during follow-up were significantly older (65.0 ± 10.4 vs 58.6 ± 11.6 years, *p* < 0.001), had a later age of onset of type 2 diabetes (54.70 ± 12.61 vs 47.61 ± 11.43 years, *p* < 0.001), a longer duration of type 2 diabetes (12.44 ± 8.89 vs 11.05 ± 6.28, *p* = 0.043) and higher MAP (98.41 ± 10.14 vs 95.54 ± 7.85 mmHg, *p* = 0.005) than those who were did not develop microvascular complications.

**Table 1:** Baseline characteristics of the study population with successfully measured Lp(a) levels

Characteristic	Cases (*n* = 1850)	*n* (%) missing data
Female sex, *n* (%)	857 (46%)	1 (0.1%)
Age (years)	65.20 (10.54)	1 (0.1%)
Age of onset diabetes (years)	54.94 (11.71)	118 (6%)
Duration of diabetes (years)	10.02 (8.40)	118 (6%)
Duration of follow-up (years)	6.97 (2.10)	11 (0.6%)
BMI, (kg/m^2^)	29.50 (27.00–33.05)	129 (7%)
HbA_1c_ (mmol/mol)	50.82 (45.46–59.57)	92 (5%)
HbA_1c_ (%)	6.80 (6.30–7.60)	92 (5%)
MAP (mmHg)	98.97 (10.80)	126 (7%)
Total cholesterol (mmol/l)	4.29 (0.93)	90 (5%)
Triacylglycerols (mmol/l)	1.43 (1.02–2.04)	92 (5%)
HDL-cholesterol (mmol/l)	1.10 (0.93–1.32)	93 (5%)
Non-HDL-cholesterol (mmol/l)	3.12 (0.90)	93 (5%)
LDL-cholesterol (mmol/l)	2.45 (0.83)	120 (6%)
Lp(a) (mg/dl)	27.40 (42.09)	0
Lp(a) (mg/dl)	11.00 (5.00–31.55)	0
Lp(a) ≥30 mg/dl, *n* (%)	483 (26%)	0
Creatinine (μmol/l)	77.00 (67.00–92.00)	121 (7%)
eGFR (MDRD) (ml min^–1^ [1.73 m]^−2^)	77.36 (22.48)	121 (7%)
Outpatient clinic patients, *n* (%)	796 (43%)	-
Smoking, *n* (%)		177 (10%)
Never	433 (26%)	
Former	943 (56%)	
Current	297 (18%)	
Microvascular complications at baseline		
Retinopathy at baseline, *n* (%)	298 (17%)	105 (6%)
Nephropathy at baseline, *n* (%)	375 (23%)	199 (11%)
Neuropathy at baseline, *n* (%)^a^	223 (31%)	1118 (60%)
Microvascular complications during follow-up		
Retinopathy during follow-up, *n* (%)	223 (16%)	156 (8%)
Nephropathy during follow-up, *n* (%)	246 (19%)	107 (6%)
Neuropathy during follow-up, *n* (%)^a^	236 (32%)	1123 (61%)

Unless stated otherwise, mean (±SD) are given for normally distributed covariates. For non-normally distributed covariates, median and IQR are given

^a^Information only available for individuals who attended an outpatient clinic (*n* = 796)

### Lp(a) and prevalent complications at baseline

In cross-sectional analyses at baseline (ESM Table 3), individuals with type 2 diabetes with Lp(a) concentrations ≥30 mg/dl did not have significantly higher ORs than those with concentrations <30 mg/dl for each of the microvascular endpoints in any of the models. These results did not change when the separate endpoints were taken together as one composite endpoint (OR 0.95, 95% CI 0.70, 1.27, *p* = 0.71). Additional analyses with Lp(a) as a continuous variable or divided into quartiles did not change these results (ESM Tables 4, 5). Furthermore, spline analyses for Model 2 did not argue for any other non-linear relationship with prevalent microvascular endpoints (ESM Fig. 1, all *p* values for deviation from linearity >0.99).

### Lp(a) and incident complications at follow-up

**Fig. 1:**
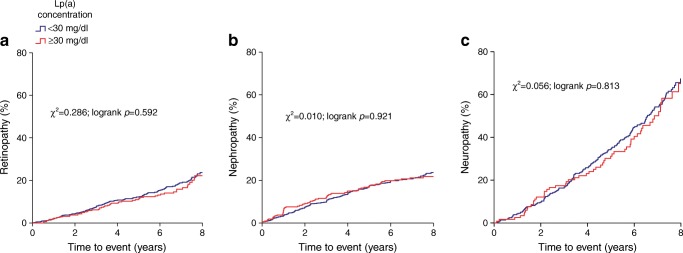
Kaplan–Meier curves for the percentage of the study population who developed retinopathy (**a**), nephropathy (**b**) or neuropathy (**c**) during follow-up according to Lp(a) level

Table 2 shows the Cox proportional hazards models of the prospective analyses. Individuals with Lp(a) concentrations ≥30 mg/dl did not have significant HRs for any of the microvascular endpoints in any of the models. Similarly, Kaplan–Meier curves created for each microvascular endpoint did not show any significant difference between the two Lp(a) concentration groups (Fig. 1). Consideration of the three microvascular endpoints together as one composite endpoint in the Cox proportional hazards regression did not change the result (HR 1.16, 95% CI 0.85, 1.60, *p* = 0.35). Additional analyses with Lp(a) as a continuous variable or divided into quartiles did not change these results (ESM Tables 4 and 5). Investigation of the relationship with kidney function defined by eGFR MDRD revealed that Lp(a) was not significantly associated with percentage eGFR MDRD decline during follow-up (ESM Table 6). Furthermore, spline analyses for Model 2 also did not show any non-linear relationship of Lp(a) concentration with incident microvascular endpoints (ESM Fig. 2, all *p* values for deviation from linearity >0.28).

**Table 2:** Hazard ratios for incident microvascular complications for patients with Lp(a) concentrations ≥30 mg/dl <30 mg/dl (reference group)

	Model 1, HR (95% CI)	*p* value	Model 2, HR (95% CI)	*p* value
Retinopathy (*n* = 183)				
≥30 mg/dl	0.94 (0.69, 1.26)	0.66	1.02 (0.73, 1.43)	0.90
Nephropathy (*n* = 185)				
≥30 mg/dl	1.04 (0.78, 1.38)	0.81	1.03 (0.73, 1.44)	0.88
Neuropathy (*n* = 202)				
≥30 mg/dl	0.99 (0.74, 1.33)	0.95	0.99 (0.70, 1.39)	0.94

*n*, numbers of patients who developed the complication

Model 1: adjusted for sex and age

Model 2: additionally adjusted for MAP, non-HDL-cholesterol, HDL-cholesterol, BMI, duration of type 2 diabetes, HbA_1c_ and smoking (never/former/current)

### LPA genotypes and Lp(a) concentrations

For the entire study population independent of microvascular complications at baseline the *LPA* SNPs rs10455872 and rs3798220 were in Hardy–Weinberg equilibrium (χ^2^ 2.20, *p* > 0.05 and χ^2^ 0.86, *p* > 0.05), respectively. Minor allele frequencies of rs10455872 and rs3798220 were 0.0648 and 0.0156, respectively (see details on genotype distribution and mean Lp(a) plasma concentration according to the carrier status of the *LPA* SNPs in Table 3). Individuals with at least one minor allele for rs10455872 and rs3798220 had significantly higher Lp(a) plasma concentrations compared with individuals with the wild-type genotype. The SNPs rs10455872 and rs3798220 explained 20% and 8% of the variation in Lp(a) levels, respectively, and jointly explained 30% of the variance.

**Table 3:** Distribution and association *LPA* SNPs with Lp(a) concentrations

SNP	*n* (%)	Mean Lp(a)	Median Lp(a)	*p* value
rs10455872				
Wild-type (AA)	1514 (87.6%)	20.95 (35.01)	9.00 (4.00–20.30)	2.35 × 10^−86^
Heterozygous (AG)	202 (11.7%)	70.58 (52.21)	62.00 (47.00–78.50)	
Homozygous (GG)	11 (0.7%)	158.76 (62.12)	167.00 (96.90–203.00)	
rs3798220				
Wild-type (TT)	1644 (96.9%)	25.23 (37.12)	11.00 (5.00–30.00)	4.33 × 10^−31^
Heterozygous (TC)	51 (3%)	94.04 (97.88)	74.95 (7.25–155.50)	
Homozygous^a^ (CC)	1 (0.1%)	74.00 (NA)	NA	

Mean (± SD), median (IQR) are given

^a^*n* = 1, SD not applicable (NA)

### LPA SNPs and incident microvascular complications

ESM Table 7 shows the Cox proportional hazards models for prospective analyses of the *LPA* SNPs, according to the additive genetic model. For both *LPA* SNPs, carriers of the minor allele did not have significant HRs for any of the microvascular complications. These results did not change when the separate microvascular endpoints were taken together as one composite endpoint (data not shown). Furthermore, these results did not change when analysing the two SNPs together as a genetic risk score (ESM Table 8).

## Discussion

In our cohort study of type 2 diabetes individuals, we did not find a cross-sectional nor a prospective association of Lp(a) plasma levels and *LPA* SNPs rs10455872 and rs3798220 with microvascular complications.

In the general population, Lp(a) is a causal risk factor for macrovascular complications [38]. The risk factor profiles associated with the development of macro- and microvascular disease in type 2 diabetes are thought to be similar [39]. If so, microvascular disease could be an extra target for the newly developed ApoA antisense therapy [40]. In our large prospective study, elevated Lp(a) concentrations were not associated either with prevalent or incident microvascular endpoints in type 2 diabetes. Our results are strengthened by the lack of association of the *LPA* SNPs rs10455872 and rs3798220, which, notably, did have their known significant effects on Lp(a) plasma levels. Moreover, in our DiaGene cohort the two SNPs rs10455872 and rs3798220 explained 20% and 8% of the variation in Lp(a) levels, respectively, and jointly explained 30% of the variance. This is relatively high compared with other type 2 diabetes populations (of about 1000 individuals), where rs10455872 explained approximately 3% of the Lp(a) variation [41]. However, in the general population, Clarke et al found that these two SNPs together explained 40% of the Lp(a) variation [12]. Overall, in our DiaGene cohort the variance explained by the SNPs is in line with the findings in the general population. Overall, our results indicate that Lp(a)-lowering therapies might not have a place in the treatment of microvascular complications in type 2 diabetes. Differences with previously reported results are unlikely to be explained by heterogeneity based on the differences between cross-sectional and prospective study designs, as we performed both and found virtually identical results. Factors that potentially contributed to the variation between the findings of the studies are ethnicity, conditions for and duration of storage of plasma samples, and method of Lp(a) measurement, which we will discuss below.

The two prospective analyses in a South Korean population by Yun et al pointed at a positive association between Lp(a) plasma levels and both diabetic retinopathy and nephropathy [19, 23]. One prospective study, on kidney function decline, not strictly confined to nephropathy, in individuals of European descent with type 2 diabetes found no association with Lp(a) levels, in line with our findings [25]. Ethnicity is associated with differences in Lp(a) plasma levels; higher levels are found in black compared with white populations, and a significant heterogeneity has been reported across Asian populations, with lower levels observed in Chinese than in Indian populations [42, 43]. These ethnic differences may be explained by genetic and environmental differences [44]. Notably, the ethnic differences in plasma levels of Lp(a) were not detectable at birth [45]. Macrovascular disease risk that associated with elevated Lp(a) levels also showed differential effects across different ethnicities [46]. However, it remains unknown whether and how ethnicity contributes to differential effects of Lp(a) on microvascular disease, ranging from no effect in individuals from European descent to increased risk in South Koreans.

Sample storage and method used to determine Lp(a) plasma concentration both influence measurements [32, 47]. Several studies have measured Lp(a) directly after venous blood withdrawal, whereas others first stored the samples in freezers, at different freezing temperatures. Kronenberg et al [47] showed that Lp(a) plasma levels decline over time when stored frozen, with a further decline observed with multiple thawing cycles. Samples stored at −20°C declined at significantly faster rate than those stored at −80°C [47]. Most of the studies so far, including the reports by Yun et al [19, 23], did not perform a KIV size-independent method, resulting in analyses with ‘biased’ Lp(a) levels [16–18, 20, 22, 24–27]. Marcovina et al [48] showed that these biases among methods of Lp(a) plasma measurement can substantially affect the outcomes. We have measured Lp(a) in previously unthawed samples (stored at −80°C) with a KIV size-independent method.

In our type 2 diabetes population, Lp(a) levels were relatively low compared with reports in the general population [13]. Individuals with type 2 diabetes are often in a hyperinsulinaemic state as a result of peripheral insulin resistance. Hyperinsulinaemia decreases Lp(a) plasma levels, as insulin suppresses ApoA production in hepatocytes at the post-transcriptional level [49]. One may speculate that, in type 2 diabetes, high Lp(a) levels do not contribute to microvascular disease risk, because of other more influential competing risk factors, such as hyperglycaemia and dyslipidaemia. However, even the highest quartiles of Lp(a) levels did not display any association with microvascular complications, suggesting that there may be no role for Lp(a) in these endpoints at all.

Finally, the KIV size remains an essential property of Lp(a). The KIV size is independently inversely associated with macrovascular disease in the general population [50]. Likewise, it could be independently associated with microvascular complications in type 2 diabetes. Unfortunately, we do not have details of the KIV size. As it is known that Lp(a) plasma levels are inversely associated with the KIV size, if this effect on microvascular disease is substantial, we would have expected to find a positive association of Lp(a) plasma levels with microvascular complications. Also, the investigated *LPA* SNP rs10455872 is strongly associated with the KIV size and showed no association with our microvascular endpoints [51]. However, we cannot exclude the possibility that the KIV size is associated with microvascular disease, and this will be the subject of ongoing studies in our population.

Strengths of our study are the prospective study design, relatively large type 2 diabetes cohort, the meticulous collection of phenotypic and risk factor data, the investigation of all microvascular endpoints and the concomitant investigation of the effects of *LPA* SNPs. Moreover, we used an immunoassay with the least Lp(a) concentration-dependent bias of the available immunoassays, and this immunoassay has a correlation coefficient (*r*) of 0.99 with values determined by the ‘gold standard’ for measuring Lp(a) (an ELISA method) [32, 47]. Furthermore, we directly stored our plasma samples in minus 80°C and used previously unthawed samples [47]. Although we performed our study with great care, we need to consider some limitations. We had 80% power to detect an HR of 1.5 for the categorical and 1.013 for the continuous analyses. All analyses in Model 2 resulted in ORs and HRs for microvascular disease very close to 1.0, with 95% confidence upper limits of around 1.5 for the categorical analyses and 1.005 for a continuous increase of 1 mg/dl. This means that true effect sizes larger than these upper limits are highly unlikely, but effect sizes within the 95% confidence intervals cannot be excluded to become significant in much larger studies. To put this into context, a 0.8% difference in HbA_1c_, as a known continuous risk factor for microvascular disease, in the Action in Diabetes and Vascular Disease: Preterax and Diamicron MR Controlled Evaluation (ADVANCE) study resulted in an HR of 1.14 (95% CI 1.03, 1.23) for microvascular disease after 5 years of follow-up [52]. This confidence interval does not contain the higher upper limit of the 95% CI in our study of HR 1.005. In even larger studies, our HR of 1.005 per 1 mg/dl increasing Lp(a) level could potentially become statistically significant and have an effect on microvascular disease risk, although this would be a very small effect. Furthermore, neuropathy data were only available for individuals who were seen as outpatients in the hospitals, so we cannot generalise our findings to the first line of care. In addition, data on KIV size are not available for our study population.

In conclusion, our data indicate that Lp(a) plasma levels do not play a major role in the development of microvascular complications in individuals from European descent with type 2 diabetes. Larger studies than ours may be able to detect very small effects of Lp(a) levels on these outcomes. Future studies taking the Lp(a) KIV size into account can further elucidate whether or not any other aspect of the Lp(a) particle is associated with the microvascular complications of type 2 diabetes.

## Electronic supplementary material

ESM (PDF 278 kb)

## Data Availability

The datasets generated during and/or analysed during the current study are not publicly available. The raw data are subject to ‘Special Categories of Personal Data (Sensitive Data)’ (GDPR, Article 9), therefore raw data sharing is not in line with the privacy principles. Also, the information provided to the participants in the study states that the individual data are only accessible to the researchers, the ethical review board and (local) authorities. The informed consent given by the participants is therefore not sufficient for open access publication of indirectly identifiable data. Datasets are available from the corresponding author upon reasonable request.

## Abbreviations

ApoA: Apolipoprotein A
EAS: European Atherosclerosis Society
Lp(a): Lipoprotein A
MAP: Mean arterial pressure
MDRD: Modification of Diet in Renal Disease
