# Supplementary material for: Lipoprotein(a) plasma levels are not associated with incident microvascular complications in type 2 diabetes mellitus
Source: Diabetologia. 2020 Mar 9;63(6):1248–57. doi: 10.1007/s00125-020-05120-9 (PMC7228906; doi:10.1007/s00125-020-05120-9)

ESM:

ESM Table 1: Post-hoc power analyses

	Power	HR
Lp(a) binary predictor (prevalent analyses)	80 %	1.4 *
Lp(a) binary predictor (prospective analyses)	80 %	1.5
Lp(a) continuous predictor (prevalent analyses)	80 %	1.09*
Lp(a) continuous predictor (prospective analyses)	80 %	1.013
rs3798220	80 %	1.7
rs10455872	80 %	1.7

*** OR**

ESM Table 2: Lp(a) median concentrations according to microvascular complication

**Baseline**	**Retinopathy (present, n=298)**	**Retinopathy (absent,n=1480)**	**P value**
Lp(a) median, IQR, (mg/dl)	10 (5.83-26.25)	11 (5-33)	0.86
	**Nephropathy (present, n=375)**	**Nephropathy (absent, n=1309)**	
Lp(a), median, IQR, (mg/dl	10 (4.7-31.4)	11.1 (5-31.75)	0.15
	**Neuropathy (present, n=223)**	**Neuropathy (absent, n=539)**	
Lp(a) median, IQR, (mg/dl)	10.90 (5.00-28.00)	11 (4.85-31.00)	0.84
**Follow-up**	**Retinopathy (present, n=223)**	**Retinopathy (absent, n=1180)**	
Lp(a) median, IQR, (mg/dl)	12 (5-35)	11 (5-32.85)	0.85
	**Nephropathy (present, n=246)**	**Nephropathy (absent, 1005)**	
Lp(a), median, IQR, (mg/dl)	11 (4.78-11)	11.25 (5-31)	0.62
	**Neuropathy (present, n=236)**	**Neuropathy (absent, n=256)**	
Lp(a) median, IQR, (mg/dl)	11 (4-29.75)	11 (5-29)	0.84

Median (IQR) are given, P values were obtained using Mann-Whitney test

ESM Table 3: Odds ratio for prevalent microvascular complications for patients with Lp(a) concentrations ≥30 mg/dL compared to those below 30 mg(dL (=reference group). n, numbers of patients who developed the complication.

	**Model 1 OR, 95% CI**	**P-value**	**Model 2, 95% CI**	**P-value**
**Retinopathy n=231**				
≥30 mg/dl	0.89 (0.66-1.19)	0.43	0.98 (0.68-1.43)	0.93
**Nephropathy n=302**				
≥30 mg/dl	1.06 (0.81-1.39)	0.66	1.11 (0.81-1.52)	0.52
**Neuropathy n=177**				
≥30 mg/dl	0.96 (0.66-1.40)	0.84	0.98 (0.64-1.50)	0.92

(n=number of events, CI; confidence intervals)

Model 1: adjusted sex and age;
Model 2 additionally adjusted MAP, Non-HDL-cholesterol, HDL cholesterol, BMI, duration of type 2 diabetes, HbA1c and smoking (never/former/current)

ESM Table 4 : OR's and HR's for microvascular complications per increasing 1 mg/dl Lp(a) concentration. n, numbers of patients who developed the complication.

	**Model 1, 95% CI**	**P-value**	**Model 2, 95% CI**	**P-value**
**Baseline**				
Retinopathy (n=231)	0.998 (0.994-1.001)	0.16	0.999 (0.995-1.004)	0.79
Nephropathy (n= 302)	0.999 (0.996-1.002)	0.67	1.000 (0.997-1.004)	0.98
Neuropathy (n= 177)	0.999 (0.995-1.003)	0.69	1.000 (0.995-1.005)	0.92
**Follow-up**				
Retinopathy (n=183)	1.000 (0.997-1.003)	0.30	1.001 (0.997-1.004)	0.72
Nephropathy (n= 185)	0.999 (0.996-1.002	0.48	0.999(0.995-1.003)	0.60
Neuropathy (n=202)	1.000 (0.996-1.003)	0.79	0.999 (0.994-1.003)	0.55

n= number of events

Model 1: adjusted sex and age
Model 2 additionally adjusted for MAP, Non-HDL-cholesterol, HDL cholesterol, BMI, duration of type 2 diabetes, HbA1c and smoking (never/former/current)

ESM Table 5 : OR's and HR's for prevalent and incident microvascular complications, respectively, by quartiles of Lp(a) concentrations concentration ≤ 25th percentile (≤5mg/dL (=reference group), >25th ≤50th percentile(>5mg/dl ≤11 mg/dl), >50th ≤75th percentile (>11 mg/dl ≤31.55 mg/dl), >75th ≤100th percentile (>31.55 mg/dl). n, numbers of patients who developed the complication.

**Baseline**	**Model 1, 95% CI**	**P-value**	**Model 2, 95% CI**	**P-value**
**Retinopathy, n=231**				
>25th ≤50th percentile (n= 86)	1.53 (1.08-2.17)	0.02	1.33 (0.86-2.06)	0.20
>50th ≤75th percentile (n=73)	1.27 (0.89-1.82)	0.19	1.12 (0.71-1.76)	0.63
>75th ≤100th percentile (n=67)	1.09 (0.76-1.57)	0.65	1.15 (0.73-1.82))	0.54
**Nephropathy, n=302**				
>25th ≤50th percentile (n=100)	1.11 (0.80-1.53)	0.55	0.95 (9.65-1.37)	0.78
>50th ≤75th percentile (n=75)	0.79 (0.56-1.11)	0.17	0.74 (0.50-1.09)	0.13
>75th ≤100th percentile (n=93)	1.02 (0.74-1.41)	0.91	1.01 (0.70-1.48)	0.94
**Neuropathy, n=177**				
>25th ≤50th percentile (n=58)	1.12 (0.72-1.76)	0.61	0.96 (0.58-1.58)	0.86
>50th ≤75th percentile (n=55)	1.11 (0.70-1.75)	0.66	0.88 (0.52-1.48)	0.62
>75th ≤100th percentile (n=53	1.02 (0.65-1.61)	0.93	0.96 (0.57-1.61)	0.88
**Follow-up**	**Model 1, 95% CI**	**P-value**	**Model 2, 95% CI**	**P-value**
**Retinopathy ,n=183**				
>25th ≤50th percentile (n=45)	0.79 (0.54-1.15)	0.22	0.66 (0.43-1.02)	0.06
>50th ≤75th percentile (n=58)	1.11 (0.77-1.58)	0.58	1.18 (0.79-1.74)	0.42
>75th ≤100th percentile (n=56)	0.90 (0.63-1.29)	0.56	0.93 (0.62-1.40)	0.73
**Nephropathy, n=185**				
>25th ≤50th percentile (n=59)	0.93 (0.66-1.32)	0.69	0.96 (0.65-1.43)	0.84
>50th ≤75th percentile (n=57)	0.87 (0.61-1.25)	0.45	0.84 (0.55-1.29)	0.43
>75th ≤100th percentile (n=63)	1.00 (0.72-1.41)	0.99	1.02 (0.68-1.52)	0.94
**Neuropathy, n=202**				
>25th ≤50th percentile (n=53)	0.93 (0.65-1.42)	0.67	0.80 (0.54-1.16)	0.24
>50th ≤75th percentile (n=57)	0.99 (0.69-1.41)	0.95	0.80 (0.55-1.17)	0.25
>75th ≤100th percentile (n=56)	0.92 (0.65-1.32)	0.66	0.80 (0.54-1.20)	0.28

Model 1: adjusted sex and age

Model 2 additionally adjusted for MAP, Non-HDL-cholesterol, HDL cholesterol, BMI, duration of type 2 diabetes, HbA1c and smoking (never/former/current)

ESM Table 6: The association of % difference eGFR with Lp(a) concentration

	Model 1 beta, 95% CI	P-value	Model 2 beta, 95% CI	P-value
**Lp(a) as binary covariate, <30mg/dl as reference, n= 1713**				
% difference eGFR MDRD (ml/min/1.73 $m^{2)}$,	-0.597 (-3.211-2.017)	0.654	-0.316 (-3,156-2.525)	0.828
**Lp(a) continuous, n= 1713**				
% difference eGFR MDRD (ml/min/1.73 $m^{2)}$,	0.005 (-0.022-0.032)	0.706	0.012 (-0.018-0.042)	0.433

n= available for analyses, CI: confidence intervals

Model 1: adjusted sex and age
Model 2 additionally adjusted for MAP, Non-HDL-cholesterol, HDL cholesterol, BMI, duration of type 2 diabetes, HbA1c and smoking (never/former/current)

ESM Table 7: Hazard ratios for microvascular complications according to the additive genetic model for rs10455872 and rs3798220, n: numbers of patients who developed the complication.

	Model 1 HR	95% CI	P-value	Model 2 HR	95% CI	P-value
**Retinopathy**						
rs10455872, n=173	0.90	0.61-1.33	0.59	1.07	0.70-1.64	0.75
rs3798220, n= 165	1.19	0.59-2.41	0.64	1.57	0.73-3.36	0.25
**Nephropathy**						
rs10455872,n=174	1.17	0.82-1.67	0.38	1.21	0.78-1.86	0.40
rs3798220, n=166	0.85	0.35-2.06	0.72	0.71	0.23-2.24	0.56
**Neuropathy**						
rs10455872, n=176	1.39	0.97-1.98	0.07	1.17	0.73-1.88	0.50
rs3798220, n=175	0.52	0.19-1.40	0.19	0.59	0.23-1.58	0.29

Model 1: adjusted sex and age;
Model 2 additionally adjusted MAP, Non-HDL-cholesterol, HDL cholesterol, BMI, duration of type 2 diabetes, HbA1c and smoking (never/former/current)

ESM Table 8: Hazard ratios for incident microvascular complications according to the continuous genetic risk score (rs10455872 and rs3798220, reference =0)

	Model 1 HR, 95% CI	P-value	Model 2 HR, 95% CI	P-value
**Retinopathy, n=163**				
Genetic risk score	0.91 (0.69-1.21)	0.52	1.20 (0.83-1.75)	0.34
**Nephropathy, n=203**				
Genetic risk score	1.16 (0.83-1.62)	0.39	1.15 (0.77-1.73)	0.49
**Neuropathy, n=172**				
Genetic risk score	1.22 (0.87-1.72)	0.25	1.03 (0.68-1.58)	0.88

Model 1: adjusted sex and age;
Model 2 additionally adjusted MAP, Non-HDL-cholesterol, HDL cholesterol, BMI, duration of type 2 diabetes, HbA1c and smoking (never/former/current)

ESM Fig. 1: Spline curves with 95 % confidence bands for the association between log-transformed Lp(a) mg/dl and OR of each prevalent microvascular endpoints. Model 1 Spline curves adjusted for sex and age; Model 2 additionally adjusted for MAP, Non-HDL-cholesterol, HDL cholesterol, BMI, duration of type 2 diabetes, HbA1c and smoking(never/former/current)

(a) Retinopathy n=231, (b) Nephropathy n=302, (c) Neuropathy n= 177 (n=number of events)

a

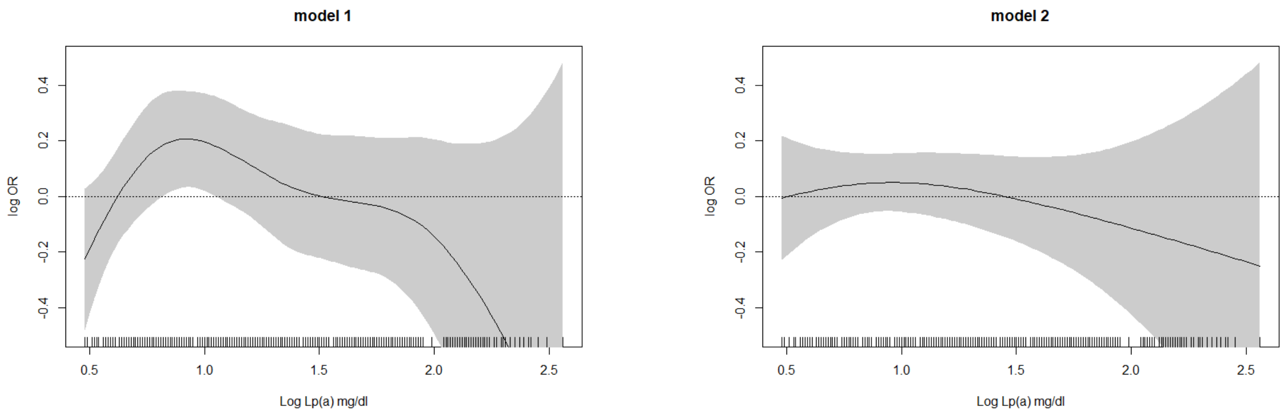

b

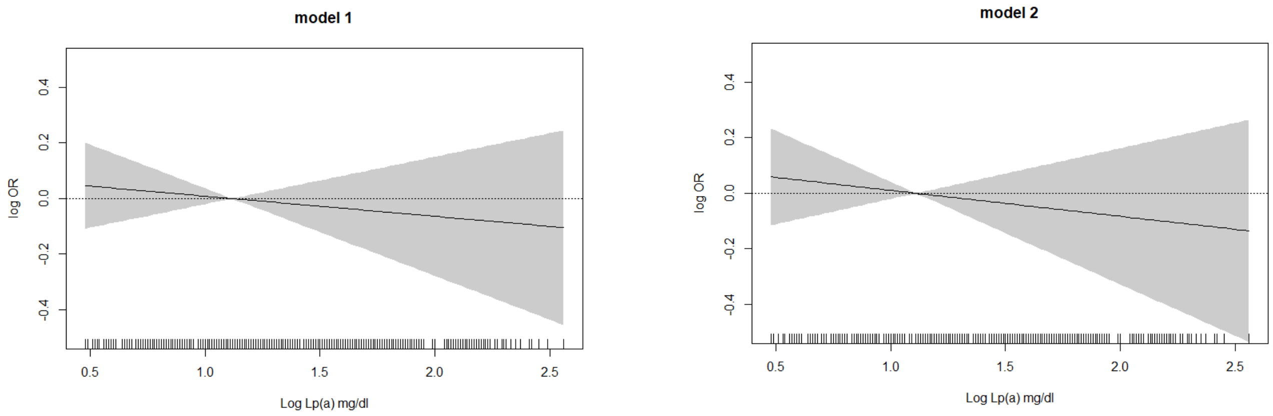

c

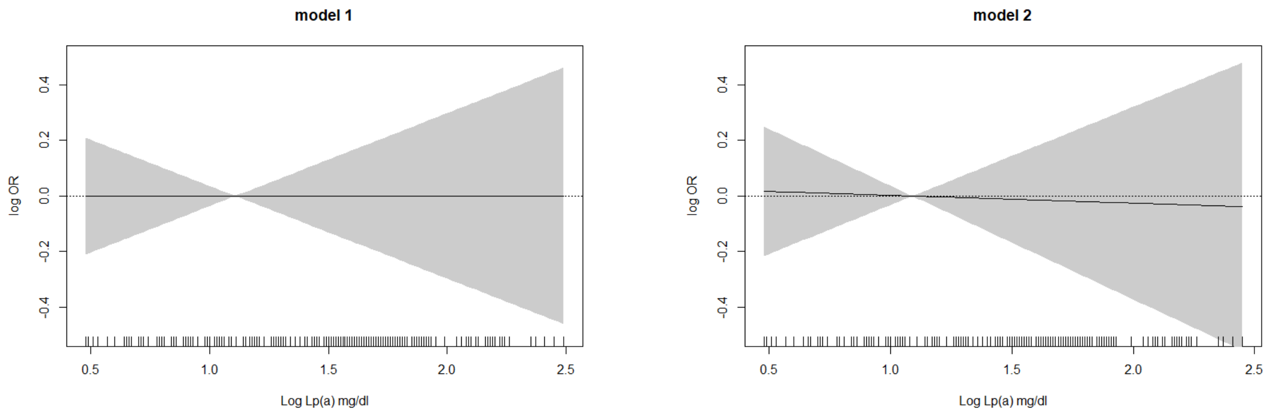

ESM Fig. 2: Spline curves with 95 % confidence bands for the association between log-transformed Lp(a) mg/dl and HR of each incident microvascular endpoints. Model 1 Spline curves adjusted for sex and age; Model 2 additionally adjusted for MAP, Non-HDL-cholesterol, HDL cholesterol, BMI, duration of type 2 diabetes, HbA1c and smoking(never/former/current)

(a) Retinopathy n=183, (b) Nephropathy n=185, (c) Neuropathy n=202 (n= number of events)

a

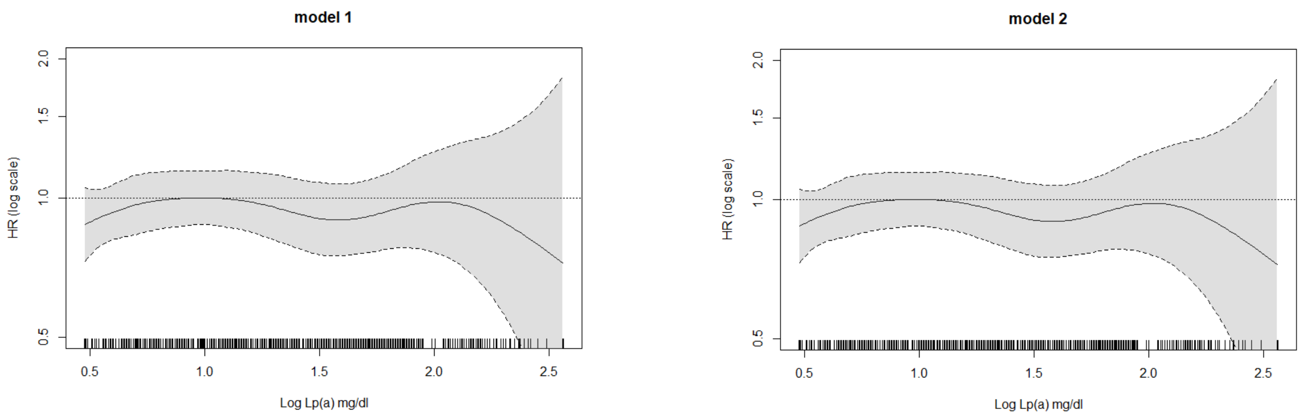

b

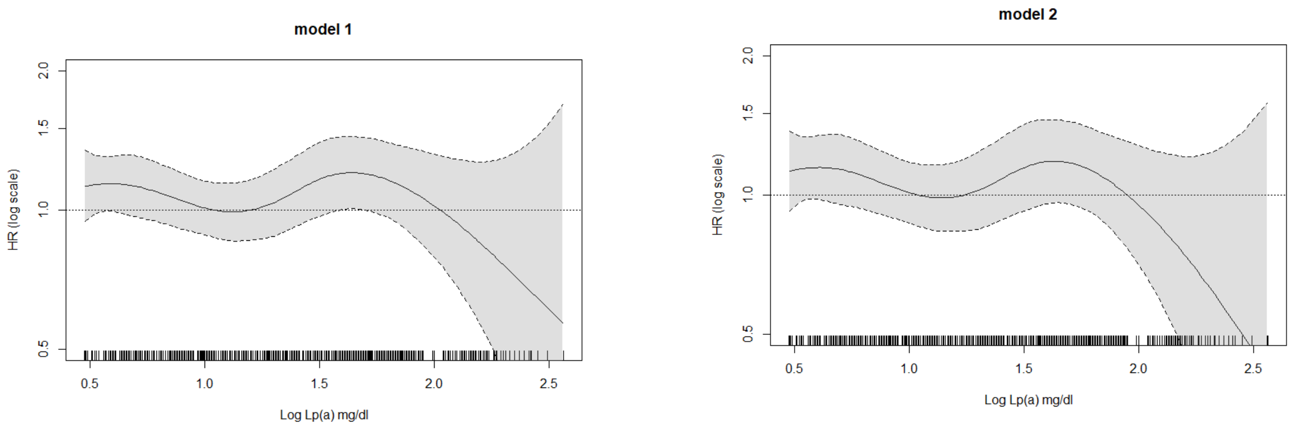

c

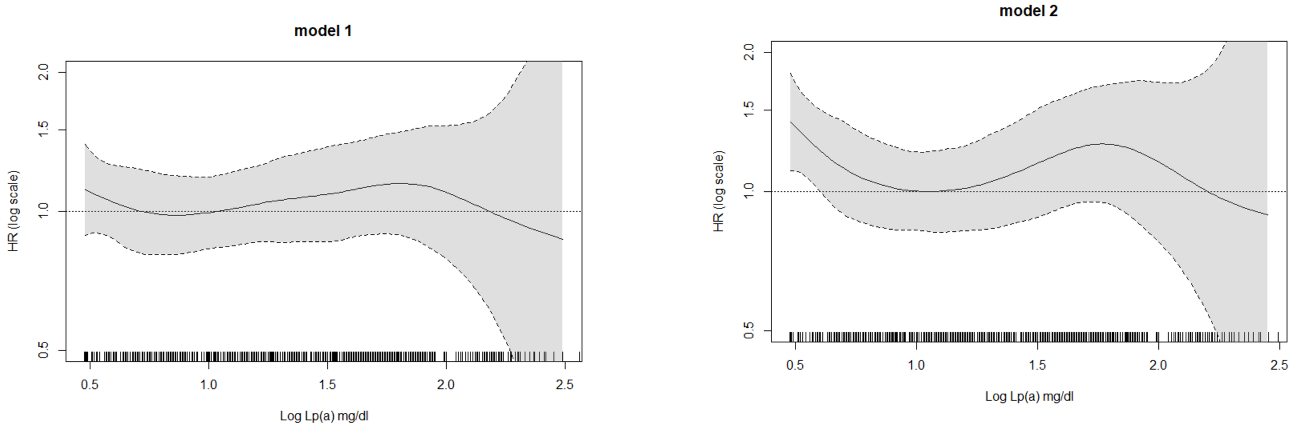

Supplement: Supplementary file 1 — (PDF 278 kb) [file 125_2020_5120_MOESM1_ESM.pdf]
